# Endoscopic score vs blood cell indices for determining timing of immunomodulator withdrawal in quiescent ulcerative colitis

**DOI:** 10.1038/s41598-019-54369-7

**Published:** 2019-11-28

**Authors:** Kazuhiro Takenaka, Keiichi Tominaga, Mimari Kanazawa, Koh Fukushi, Takanao Tanaka, Akira Kanamori, Takeshi Sugaya, Kouhei Tsuchida, Makoto Iijima, Kenichi Goda, Atsushi Irisawa

**Affiliations:** 1 Department of Gastroenterology, Dokkyo Medical University, Tochigi, Japan; 2 Department of Internal Medicine, Japanese Red Cross Ashikaga Hospital, Tochigi, Japan

**Keywords:** Ulcerative colitis, Colonoscopy

## Abstract

While immunomodulators (IMs) are used as key drugs in remission maintenance treatment for ulcerative colitis (UC), there has been no evidence to date for determining monitoring methods and drug withdrawal. Therefore, we examined if a decrease in white blood cell count (WBC) and an elevation in mean cell volume (MCV) could be used as optimization indices and if mucosal healing (MH) could be a rationale for determining the time of IM withdrawal. Subjects were 89 UC patients who were using IMs and for whom clinical remission had been maintained. Those with a Rachmilewitz Clinical Activity Index score of 4 or lower and those with a Mayo endoscopic subscore (MES) of 0 or 1 were defined as MH. The remission maintenance rates of the following comparative groups were examined: an IM continuation group and an IM withdrawal group; an IM continuation group with a WBC of less than 3000 or a MCV of 100 or greater and an IM continuation group with a WBC of 3000 or greater and a MCV of 99 or lower; an IM continuation group of patients for whom MH had been achieved and an IM continuation group of patients for whom MH had not been achieved; and an IM withdrawal group with a MES of 0 and an IM withdrawal group with a MES of 1. A significantly higher remission maintenance rate was observed in the IM continuation group compared to the withdrawal group (p < 0.01). No significant difference was observed between the IM continuation group with a WBC of less than 3000 or a MCV of 100 or greater and the IM continuation group with a WBC of 3000 or greater and a MCV of 99 or lower (p = 0.08). Higher remission maintenance rates were observed in the IM continuation group of patients for whom MH had been achieved compared to the IM continuation group of patients for whom MH had not been achieved (p = 0.03). No significant difference was observed between the IM withdrawal group with MES 0 and the IM withdrawal group with MES 1. (p = 0.48). This retrospective study showed that remission maintenance could be firmly obtained by continuing IM administration in case of endoscopic MH; however, MH was not an indicator of IM withdrawal.

## Introduction

Ulcerative colitis (UC) is a chronic inflammatory bowel disease (IBD) of unknown etiology. The disease presents with symptoms such as abdominal pain, bloody stool, diarrhea, and weight loss and is characterized by repeated relapse and remission. Because no fundamental therapeutic strategies have been established, the goal of treatment is generally to maintain the remission phase for a long period after remission induction therapy^1,2^. However, remission maintenance is often difficult. Recently, achievement of mucosal healing (MH) has been recognized as an important factor for remission maintenance, and shown to yield a low relapse rate, reduce hospitalization, result in a low rate of conversion to surgical treatment, reduce healthcare costs, and improve the quality of life of patients^3–12^.

Immunomodulators (IMs), primarily azathioprine, are recommended by guidelines in various countries for the treatment of UC patients who are steroid-dependent or experiencing difficulty with steroid withdrawal^13–17^. It has been found that the continued oral administration of IMs following remission may lead to a high remission maintenance rate that exceeds 50% and a large number of reports have stated that IMs are significantly efficacious at the time of steroid dose-reduction and remission maintenance^18–20^. Meanwhile, the optimal dosage of IM varies greatly between individual patients due to variation in metabolic disposition and when IMs are administered. Attention needs to be paid to genetic hair loss, severe myelosuppression, dose- and metabolism-dependent hepatic dysfunction, nausea and fatigue, lymphoproliferative disease resulting from long-term administration, and nonmelanoma skin cancer (NMSC)^21–24^. An onset of malignant disease as a result of the long-term use of IMs is a serious issue, hence, it is necessary to monitor the condition of UC and consider IM withdrawal where necessary. However, it has been reported that relapse rates following IM withdrawal are high, and there is a dilemma between the advantage of withdrawal and the risk of relapse^25,26^.

With regard to rheumatoid arthritis, the “treat-to-target” strategy and the concept of “tight control and disease monitoring” have long been recognized; they have also been increasingly accepted in the area of IBD. To date, there have been reports on an investigation into the selection of treatment methods based on endoscopic monitoring for a case of Crohn’s disease (CD) requiring surgical treatment, and an investigation into the dose reduction of 5-aminosalicylic acid (5-ASA) preparations due to endoscopic mucosal healing (MH). Many of these reports suggested that achieving MH is the goal of IBD treatment and that the degree of MH can be an index for selecting a treatment method^12,27–29^. MH is the obvious optimal target for IBD treatment, and there are many studies on treat to target to achieve the treatment goal^28,30,31^. On the other hand, there are no randomized controlled trial on treatment withdrawal or drug dose adjustment based on MH. Therefore, as the research gap, in UC cases that achieved MH remains a clinical problem as to whether IMs withdrawal is possible or not. MH may also be an index for IM withdrawal in UC treatment. However, there has been no investigation into whether MH can be an index for IM withdrawal. The aim of the present study was to examine if MH, which is currently considered the goal of UC treatment, can be a rationale for IM withdrawal in UC cases where long-term remission has been achieved.

## Results

### Patient’s characteristics

The backgrounds of 89 subjects are shown in Table 1. The mean age was 44 years (14–18 years), and there were 47 males (52.8%) and 42 females (47.2%). Of these, 25.8% (n = 23) were suffering from left-sided UC and 74.2% (n = 66) were suffering from total UC. An endoscopic assessment observed a Mayo endoscopic subscore (MES) of 0 in 37 cases (41.6%), a MES of 1 in 42 cases (47.2%), and a MES of 2 in 10 cases (11.2%). The number of IM withdrawal cases was 33 (37.1%).

**Table 1:** Characteristics of patients eligible for analysis.

Mean age (range)	44.0 (14–81)
Female/Male	42/47
Extent of lesion (pancolitis/left-sided colitis/proctitis)	66/23/-
Disease type (relapse-remitting/chronic continuous/first attack)	83/9/-
Duration of IM treatment (weeks)	48.7
MES (0, 1, 2)	37/42/10
Mean WBC (/*µ*L)	5250
Mean MCV	93.6
IM continued/IM withdrawn	56/33

### The inter-rater agreement on endoscopic findings

Inter-rater agreement (multi-rater kappa statics) for the endoscopic findings (MES) was performed. Regarding the agreement rate of endoscopic evaluation between the 2 endoscopists, a high Cohen’s kappa coefficient (κ = 0.75) was obtained, indicating virtually complete agreement.

### Remission maintenance in patient with IM continued or IM withdrawal

**Figure 1:**
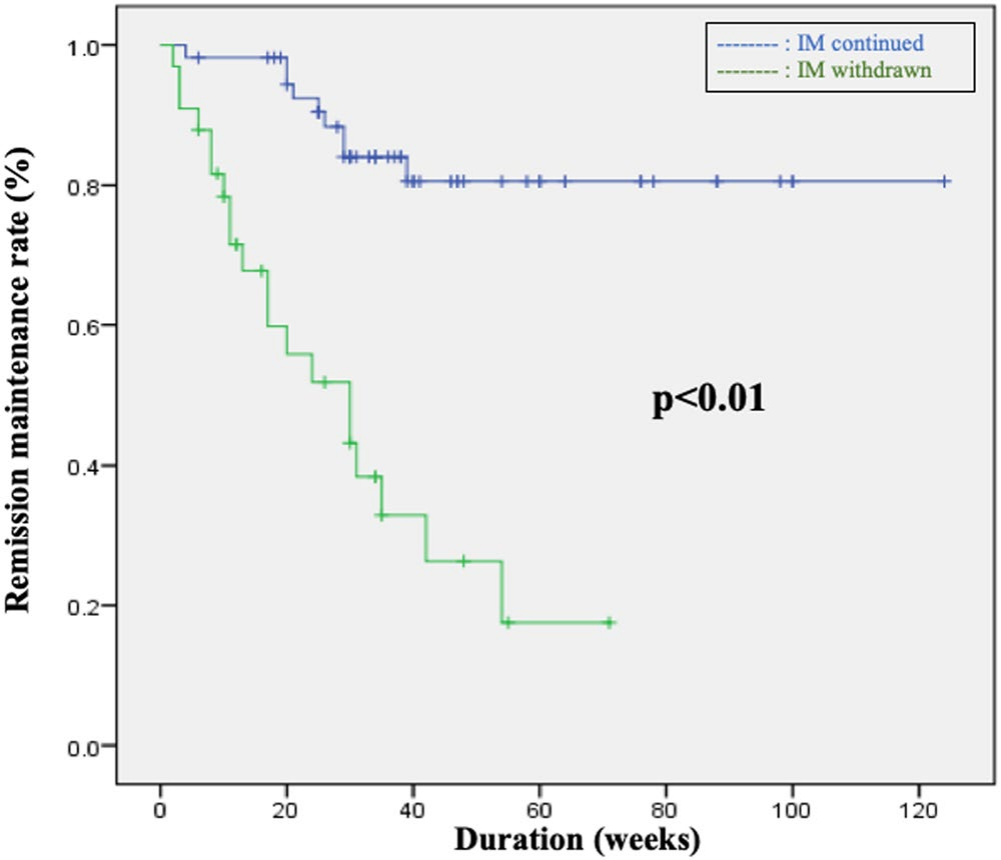
Comparison between patients in whom IMs were withdrawn and continued groups revealed a significant difference in remission maintenance rates (p < 0.01), which was the secondary endpoint.

**Figure 2:**
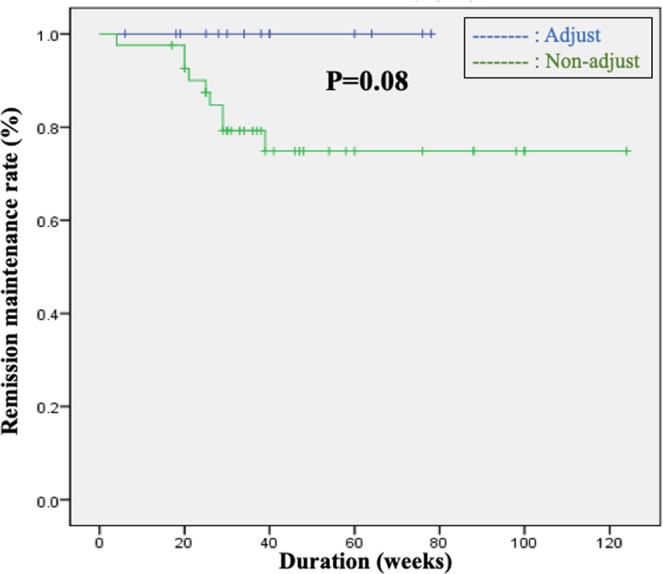
Comparison between the IM Adjust and Non-Adjust groups in patients with continued use of IMs revealed a significant difference in remission maintenance rates (p = 0.08), which was the secondary endpoint.

Firstly, we comparatively evaluated the rate of remission maintenance between the IM continued and IM withdrawal groups. Although there was no significant difference in the background of the patients between the groups (Table 2), the rate of remission maintenance was higher in the IM continued group than in the withdrawal group (*P* < 0.01) (Fig. 1). In addition, in patients where IM was continued, there was no significant difference in the rate of remission maintenance between the adjusted and non-adjusted patient groups (Table 3, Fig. 2).

**Table 2:** Comparison of patient characteristics between the IM continued and IM withdrawn groups.

	IM continued	IM withdrawn	P value
	n = 56	n = 33	
Mean age	44.0	43.9	NS
Female/Male	30/26	17/16	NS
Extent of lesion (pancolitis/left-sided colitis/proctitis	45/11/-	21/12/-	NS
Disease type (relapse-remitting/chronic continuous/first attack)	50/6/-	33/3/-	NS
Duration of IM treatment (weeks)	57.4	40.2	NS
MES (0, 1, 2)	22/26/8	15/16/2	NS
Mean WBC (/*µ*L)	5270	5230	NS
Mean MCV	93.4	93.7	NS

**Table 3:** Comparison of patient characteristics in IM continuous group.

	Adjust	Non-Adjust	P value	MH	non-MH	P value
	n = 41	n = 15		n = 48	n = 8	
Mean age	47.6	47.3	NS	43.9	43.9	NS
Female/Male	19/23	7/7	NS	23/25	3/5	NS
Extent of lesion (pancolitis/left-sided colitis/proctitis)	32/9/-	13/2/-	NS	38/9/-	7/2/-	NS
Disease type (relapse-remitting/chronic continuous/first attack)	37/4/-	13/2/-	NS	42/6	8/-/-	NS
Duration of IM treatment	48.0	48.8	NS	44.9	45.2	NS
Mean WBC (/*µ*L)	—	—	—	5270	5400	NS
Mean MCV	—	—	—	93.4	93.8	NS
MES (0, 1, 2)	15/20/6	7/6/2	NS	—	—	—

### Remission maintenance in the view point of endoscopic MH

**Figure 3:**
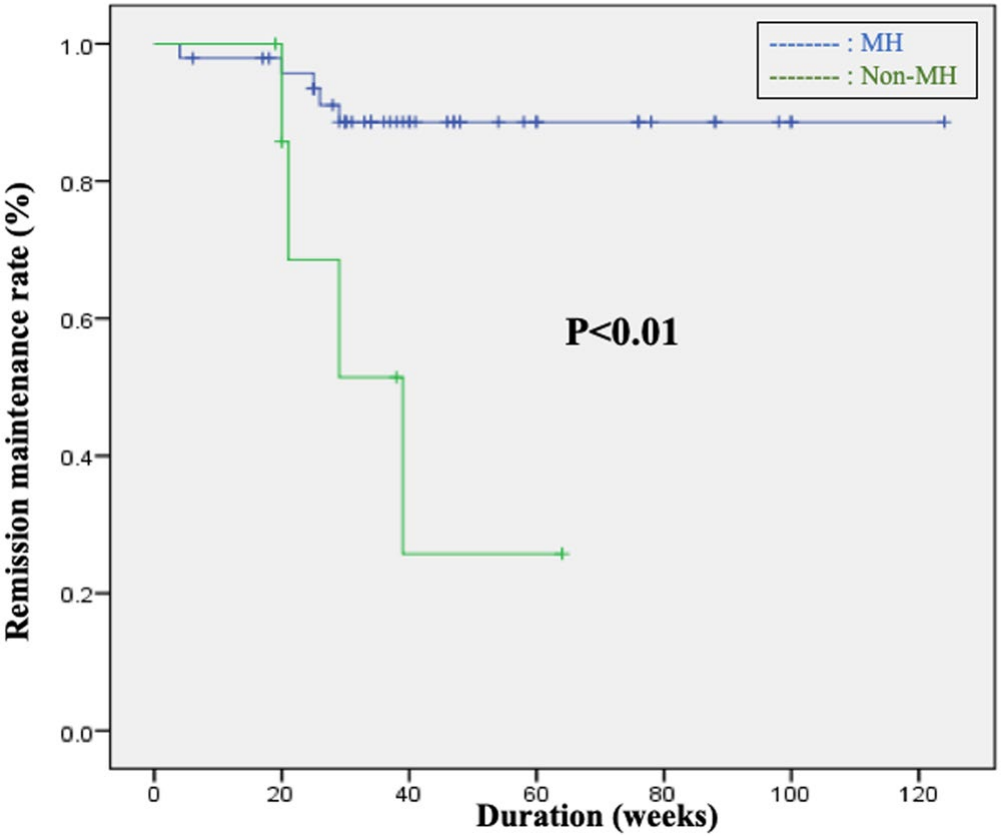
Comparison between the MH and non-MH groups in patients with continued use of IMs revealed a significant difference in remission maintenance rates (p = 0.01), which was the secondary endpoint.

**Figure 4:**
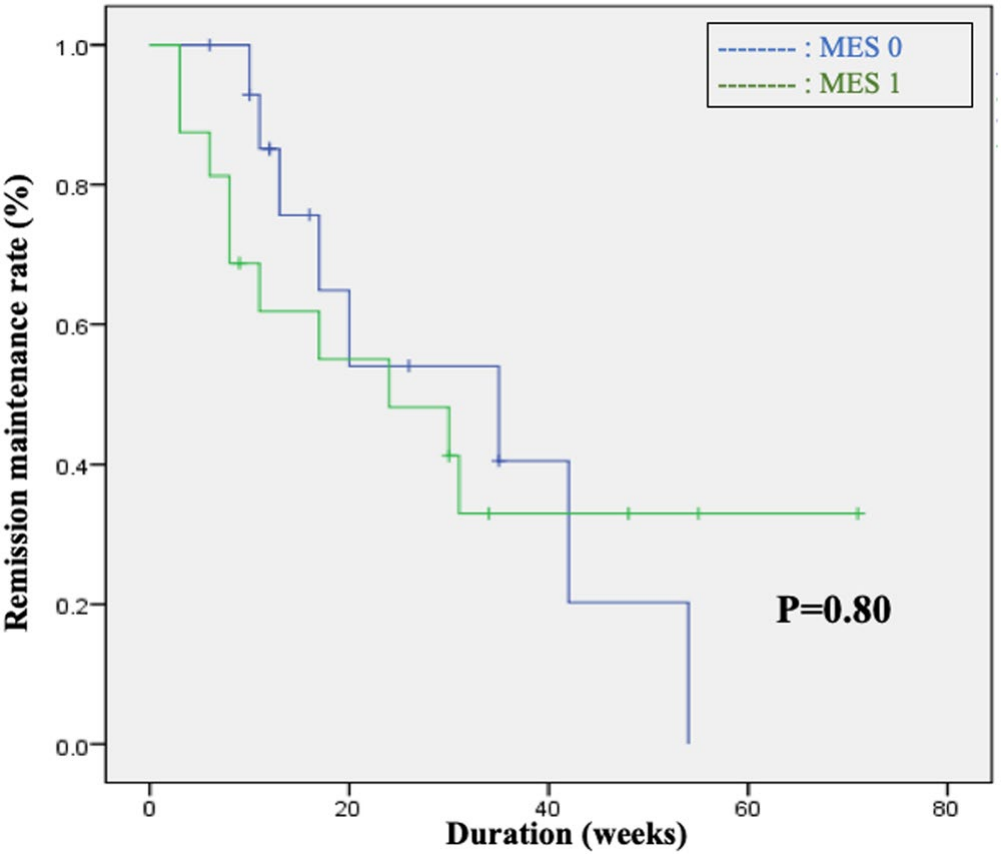
Comparison between the MES 0 and MES 1 groups among patients in whom IMs were withdrawn revealed no significant difference in remission maintenance rates (p = 0.8), which was the primary endpoint.

Within the IM continuation group that included 56 cases, a comparative analysis of remission maintenance rates was performed between the MH group (MES 0, 1) and the non-MH group (MES 2). While no significant difference was observed between the 2 groups in terms of patient backgrounds (Table 3), remission maintenance rates in the MH group were significantly higher than those in the non-MH group (*p* < 0.01) (Fig. 3). In other words, the analysis found that once MH is achieved, it is possible to maintain remission at a satisfactory level by continuing IMs; however, the continuation of IMs gives rise to problematic adverse events. Considering this viewpoint, we subsequently evaluated remission maintenance after withdrawal of IM in patients where MH had been achieved. In the IM withdrawal group that included 33 cases, 31 had IM withdrawn after MH was confirmed by endoscopy; however, remission maintenance rates in these cases were clearly lower than those of the IM continuation group. Moreover, a comparative analysis within the IM withdrawal group by pre-withdrawal MES (MES 0, MES 1) observed no significant difference in remission maintenance rates between the groups (*p* = 0.80) (Table 4, Fig. 4).

**Table 4:** Comparison of patient characteristics between the MES 0 and MES 1 groups.

	MES 0	MES 1	P value
	n = 15	n = 16	
Mean age	43.9	43.8	NS
Female/Male	8/7	7/9	NS
Extent of lesion (pancolitis/left-sided colitis/proctitis)	11/7/-	8/5/-	NS
Disease type (relapse-remitting/chronic continuous/first attack)	15/-/-	16/-/-	NS
Duration of IM treatment (weeks)	41.9	40.5	NS
Mean WBC (/*µ*L)	5200	5280	NS
Mean MCV	93.5	93.3	NS

## Discussion

In treating UC cases with repeated relapses and remissions, IMs as well as 5-ASAs play a significant role in maintaining remission. Specifically, IMs are suitable for treatment of steroid-dependent UC cases where maintenance has not been achieved through 5-ASA monotherapy^20^. IMs used to treat UC include thiopurine agents and methotrexates, and guidelines in Japan, the US, and Europe recommend the use of these drugs when indicated^14–18^. Unfortunately, methotrexates are not funded through the Japanese national health insurance scheme; therefore, only thiopurine agents are used. However, a large number of reports have shown the efficacy of methotrexates^32,33^. Additionally, it has been suggested that, apart from their use for maintaining remission in UC and CD cases, the concomitant administration of IMs with infliximab suppresses the immunogenicity of infliximab^34,35^.

On the other hand, there are concerns that the long-term administration of IMs may cause myelotoxicity, liver toxicity, infections, and malignancies (lymphoproliferative disorders or NMSC)^22–24,36,37^. According to a prospective observational cohort study from France, the incidence rates of Hodgkin’s lymphoma and non-Hodgkin lymphoproliferative disorder were 0.90 per 1000 patient-years in IM receiving, 0.20 per 1000 patient-years in those who had discontinued, and 0.26 per 1000 patient-years in those who had never received IM. The multivariate-adjusted hazard ratio of lymphoproliferative disorder between patients receiving IM and those who had never received the drugs was 5.28. Median follow-up period of this study was 35 months^24^. In order to avoid these IM-induced adverse events, it is necessary to consider withdrawing IM treatment; however, IM withdrawal may exacerbate UC symptoms. In fact, there have been a number of reports which show high relapse rates in UC cases following IM withdrawal^22–24^. Moreno-Rincón *et al*.^26^ reported that the relapse rate within 1 year of IM withdrawal in UC cases where clinical remission had been maintained was 18.88%, and that the rate within 4 years was 40.67%. Furthermore, Fraser AG *et al*.^38^ reported that in UC cases where clinical remission had been observed, the proportion of patients for whom remission could be maintained following IM withdrawal was 25.2%. In other words, it was suggested that clinical remission may not constitute a rationale for IM withdrawal. Meanwhile, due to the development of biologics and the advancement of endoscopic devises in recent years, the goal of UC treatment is shifting from clinical remission to MH. In fact, a large number of studies have reported that achieving MH leads to a decrease in relapse rates and the likelihood of subsequent surgical treatment^3,7,8,29,39^. In this way, the clinical significance of IMs is widely recognized; however, no investigation into IM withdrawal based on MH has been conducted. The present study is the first report on IM withdrawal based on MH.

In recent years, the MES has been widely used in MH evaluation. The present investigation also employed the MES in assessing MH. In the present investigation, even though IMs were withdrawn following the achievement of a MES of 0–1, the results showed that remission maintenance rates within 3 and 5 years of withdrawal were low. These results suggested that MH findings can be referred to in determining if IMs should be withdrawn or not. We consider that if the disease activity is so high in a UC patient that they are administered IMs, *i.e*. a UC patient presenting with steroid-dependency, the initial level of disease activity is high. Hence, IMs will be required for remission maintenance. This may explain why the disease easily relapses following IM withdrawal. We have previously reported that the dosage of 5-ASAs can be reduced if MH is achieved (particularly in cases where the MES is 0). That said, while it is possible to manage the administration of 5-ASAs in anticipation of dose reduction, in other words, while it is possible to perform the maintenance of administration of low-dose 5-ASAs, in managing IM administration, withdrawal instead of dose reduction is anticipated. Therefore, it is considered that the association of 5-ASAs with a MES of 0 (the finding that a MES of 0 may be used as an index for the dose reduction of 5-ASAs) is different from the results of the present study. Additionally, while it has been reported that there are differences between MES 0 and MES 1 in terms of remission maintenance rates and surgery rates^2,40–42^, we found that it is also important to not only perform an endoscopic mucosal assessment but also to confirm histological healing, in order to ensure remission maintenance^43^. As the research gap in UC treatment, we studied the clinical issue of IM withdrawal was possible or not in UC cases that achieved MH. However, UC cases that achieve MH or even if MES 0, withdrawal of IMs was made relapse easily. This result indicates that UC cases using IMs are difficult to maintain remission after IMs withdrawal even if MH was achieved. Thus, the achievement of MH is not considered to be a criterion for withdrawal of IMs. It can be considered that examining relapses following IM withdrawal from a perspective of whether histological healing has been achieved or not may provide different results. Further investigations are required in this aspect.

Meanwhile, remission maintenance rates in the IM continuation group were higher than those in the IM withdrawal group. As this result is consistent with existing reports, it is suggested that the patient population of the present study was receiving optimal and appropriate treatment with IMs. We consider that the data underpin the accuracy of the results for the primary endpoint of the present study and are important. Also, while the present investigation suggested that achieving endoscopic MH may lead to sufficient remission maintenance as long as IMs are continued, we consider that this indicates that simply using IMs will not lead to remission maintenance. In other words, it is necessary to appropriately use IMs in patients who have continued to be orally administered IMs, by assessing treatment effects through regular endoscopic observations and the monitoring of CRP, calprotectin, and occult blood. In the present study, the relapse rate in the group of patients for whom IMs were adjusted by white blood cell count (WBC) and mean cell volume (MCV) was 0%. It is generally recommended that IM treatment is adjusted by measuring 6-thioguanine nucleotide and thiopurine methyltransferase activities. However, this approach is not practical in the actual clinic for the following reasons: there is deviation in values due to the measurement methods; measurement values are not readily available; and these measurements are not funded through the Japanese national health insurance scheme^13,44^. The results of the present study suggested that determining the applicability of IMs based on WBC and MCV is effective.

The limitations of the present study are: firstly, it was a single-center retrospective study; secondly, the number of cases was small; and thirdly, centralized reading was not adopted for endoscopic observation, and there is discrepancy between assessors to a certain extent. However, since the Cohen’s kappa coefficient for inter-rater agreement in the present study was high at 0.75, we considered that the level of objectiveness was maintained to a certain extent in relation to the above-mentioned third limitation. It would have been ideal if the present study had been a prospective randomized control trial (RCT); however, the study involved drug withdrawal, and it was difficult to conduct the study as a prospective RCT. Given the limitations of the present study, we believe that the results provided important insights for developing UC treatment strategies going forward.

In conclusion, this retrospective study showed that remission maintenance could be firmly obtained by continuing IM administration in case of endoscopic MH; however, MH was not an indicator of IM withdrawal.

## Methods

### Study design

The present study is a retrospective cohort study conducted in 2 medical institutions and approved by the ethics committee of each institution, Dokkyo Medical University Hospital and Japanese Red Cross Ashikaga Hospital (approval no. R-22-3J). This study was conducted in accordance with the ethical principles associated with the Declaration of Helsinki and registered in the University Hospital Medical Network Clinical Trials Registry [UMIN000036506]. The option to opt-out of the study was communicated to the patients via our website with the following message. “Dokkyo Medical University Hospital and Japanese Red Cross Ashikaga Hospital now conducting research using medical data from patients who treated for ulcerative colitis. There will be no additional effort on patients for conducting this study. In addition, we will conduct research in compliance with laws and regulations regarding the protection of patient privacy. If you do not want your medical data to be used in this study, please contact your doctor.”

The primary endpoint was the remission maintenance rate following IM withdrawal indicated by a MES of 0. Secondary endpoints were remission maintenance rates through continued IM administration, remission maintenance rates in an IM continuation group where MH had been achieved, and remission maintenance rates in an IM continuation group where adjustments were made.

### Patients

To select eligible patients, we retrospectively reviewed the medical records of 283 UC patients aged between 14 and 81 years who were treated at Dokkyo Medical University Hospital and Japanese Red Cross Ashikaga Hospital between April 2010 and March 2018. Of the 283 cases, a case where remission had not been achieved within 1 year of the oral administration of IMs and a case with a history of anti-TNFα antibody agent administration were excluded. Moreover, in selecting patients, the following definitions were adopted: clinical remission was defined as a Rachmilewitz Clinical Activity Index score of 4 or lower; MH was defined as MES 0 or 1; and the IM Adjust group was to consist of patients with WBC > 3000 (µL) or MCV ≤ 100^45^.

Regarding administration of 5-ASA, the following 5-ASA preparations were used: a time-dependent ASA preparation (Pentasa®, 2000–4000 mg/d; Kyorin Pharmaceutical Co., Ltd., Tokyo, Japan), a pH-dependent ASA preparation (Asacol®, 2400–3600 mg/d; Zeria Pharmaceutical Co., Ltd., Tokyo, Japan), a pH-dependent MMX^®^ ASA (Lialda®, 2400–4800 mg/d; Mochida Pharmaceutical Co., Ltd., Tokyo, Japan), and salazosulfapyridine (Salazopyrin®, 2000–4000 mg/d; Pfizer Japan Inc., Tokyo, Japan), which are approved in Japan. Regarding IM preparations, azathioprine (Imuran®, 25–75 mg/d; Aspen Japan K.K., Tokyo, Japan) was administered.

### Endoscopic evaluation

According to the Montreal Classification of UC, the colon was divided into the following 3 segments^46,47^: (1) Ulcerative proctitis (E1) (Proctitis type): involvement limited to the rectum (i.e., proximal extent of inflammation is distal to the rectosigmoid junction). (2) Left-Sided UC (E2) (Left-sided type): involvement limited to the portion of the colorectum distal to the splenic flexure. (3) Extensive UC (E3) (Pancolitis type): involvement extends proximal to the splenic flexure.

**Figure 5:**
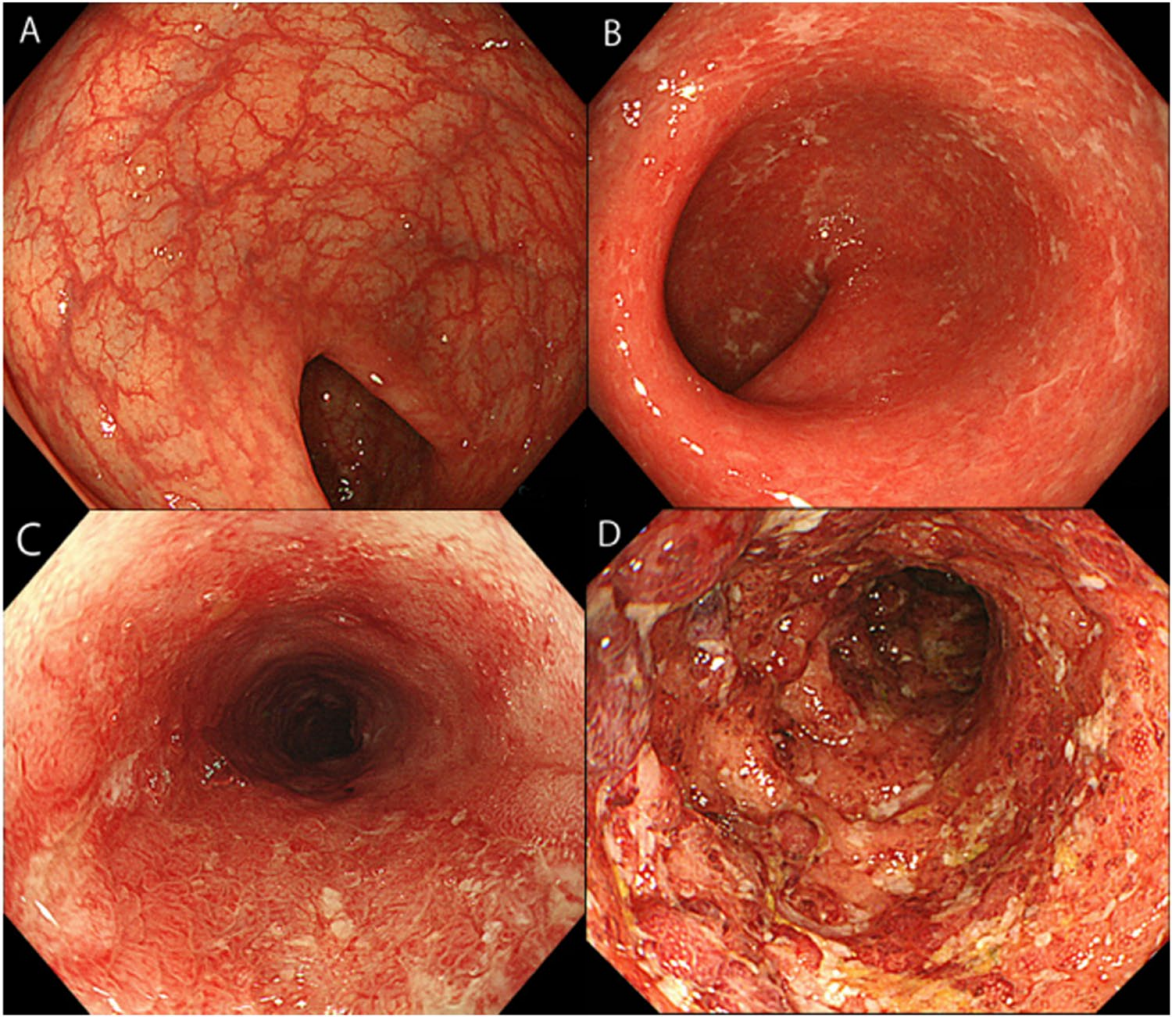
Endoscopic image of Mayo endoscopic subscore (MES). (**A**) Endoscopic image of MES 0 (no friability and granularity and intact vascular pattern). (**B**) Endoscopic image of MES 1 (mild erythema or decreased vascular pattern). (**C**) Endoscopic image of MES 2 (marked erythema, absent vascular pattern, friability, and erosions). (**D**) Endoscopic image of MES 3 (spontaneous bleeding and ulceration).

Endoscopic evaluation was based on the MES^29,45^, where a MES of 0 (no friability, granularity, and intact vascular pattern) corresponded to normal mucosa and a MES of 1 (mild erythema or decreased vascular pattern) corresponded to healed mucosa. As previously mentioned, MH was defined as a MES of 0 or 1. In addition, a MES of 2 (marked erythema, absent vascular pattern, friability, and erosions) and a MES of 3 (spontaneous bleeding and ulceration) were regarded to correspond to mucosa in the active phase (Fig. 5). Endoscopic findings obtained after at least 1 year of clinical remission were evaluated by 2 endoscopists. Each was selected from the 2 medical institutions from those with 10 or more years’ experience and who specialized in IBD, annually treating 200 or more patients with IBD. Endoscopic findings on which the 2 endoscopists agreed were included in analyses. When they disagreed on the evaluation of the findings, cases were scored by assigning 1 point to each of the following 3 items: presence of redness, visible vascular pattern, and fine mucosal granularity. When the mean scares calculated by the 2 endoscopists was 2 or higher, patients were determined to have a MES score of 1. To evaluate endoscopic findings, the scores of the colon segments with the most severe inflammation were used.

### Statistical analyses

Statistical analyses were performed with IBM SPSS Statistics 24® (IBM Japan, Ltd., Tokyo, Japan). The Cohen’s kappa coefficient (κ) was calculated to determine the agreement rate between the 2 endoscopists who evaluated the endoscopic findings. The Pearson χ^2^ test was performed to compare sex, affected area, endoscopic classification, histological classification, and smoking rate. When the expected value was <5, the Fisher exact test was performed. The Mann-Whitney U test was performed to compare mean age and mean disease duration. To compare the remission maintenance rate, survival curves were generated using the Kaplan-Meier method and the log-rank test was performed. The Cox proportional hazards model was used to identify predictors of clinical relapse. *P* < 0.05 indicated statistical significance.

## Data Availability

The database used for statistical analysis that provided data used to support the findings of this study are restricted by the Hospital Ethical Board in order to protect patient privacy. Data are available for researchers who meet the criteria for access to confidential data. More information is available from Keiichi Tominaga, MD, tominaga@dokkyomed.ac.jp.
